# Preference criteria for regorafenib in treating refractory metastatic colorectal cancer are the small tumor burden, slow growth and poor/scanty spread

**DOI:** 10.1038/s41598-021-94968-x

**Published:** 2021-07-28

**Authors:** Hung-Chih Hsu, Kuo-Cheng Huang, Wei-Shone Chen, Jeng-Kai Jiang, Shung-Haur Yang, Huann-Sheng Wang, Shih-Ching Chang, Yuan-Tzu Lan, Chun-Chi Lin, Hung-Hsin Lin, Sheng-Chieh Huang, Hou-Hsuan Cheng, Tsai-Sheng Yang, Chien-Chih Chen, Yee Chao, Hao-Wei Teng

**Affiliations:** 1 Division of Hematology-Oncology, Chang Gung Memorial Hospital at Linkou, Tao-Yuan City, Taiwan; 2 College of Medicine, Chang Gung University, Tao-Yuan City, Taiwan; 3 Department of Hematology and Medical Oncology, Koo Foundation Sun Yat-Sen Cancer Center, Taipei, Taiwan; 4 School of Medicine, National Yang-Ming University, Taipei, 112 Taiwan; 5 School of Medicine, National Yang Ming Chiao Tung University, No. 155, Sec. 2, Li-Nong Street, Taipei, 112 Taiwan; 6 Department of Surgery, Division of Colon and Rectal Surgery, Taipei Veterans General Hospital, Taipei, 112 Taiwan; 7 Department of Surgery, National Yang Ming Chiao Tung University Hospital, Yilan, Taiwan; 8 Department of Surgery, Koo Foundation Sun Yat-Sen Cancer Center, Taipei, Taiwan; 9 Department of Oncology, Division of Medical Oncology, Taipei Veterans General Hospital, No. 201, Sec. 2, Shih-Pai Road, Taipei, 112 Taiwan

**Keywords:** Cancer, Cancer therapy, Gastrointestinal cancer

## Abstract

Given the unclear preference criteria for regorafenib in treating refractory metastatic colorectal cancer (mCRC), this study aimed to construct an algorithm in selecting right patients for regorafenib. This was a multicenter retrospective cohort study. Patients with pathology confirmed mCRC and administered with regorafenib for > 3 weeks were enrolled. Patients with good response were defined to have progression-free survival (PFS) of ≥ 4 months. The Kaplan–Meier plot was used to analyze survival. A Cox proportional hazards model was used to analyze univariate and multivariate prognostic factors and was visualized using forest plot. A clustering heatmap was used to classify patients according to responses. The decision tree and nomogram were used to construct the approaching algorithm. A total of 613 patients was analyzed. The median PFS and overall survival (OS) were 2.7 and 10.6 months, respectively. The partial response and stable disease rate are 2.4% and 36.4%. The interval between metastasis (M1) and regorafenib, metastatic status (number, liver, and brain), and CEA level were independent prognostics factors of PFS that classifies patients into three groups: good, bad and modest-1/modest-2 group with PFS >  = 4 months rates of 51%, 20%, 39% and 30%, respectively. Results were used to develop the decision tree and nomogram for approaching patients indicated with regorafenib. The preference criteria for regorafenib in treating patients with refractory mCRC are small tumor burden (CEA), slow growth (interval between metastasis and regorafenib) and poor/scanty spread (metastatic status: number and sites of metastasis): The 3S rules.

TRIAL registration ClinicalTrials.gov Identifier: NCT03829852; Date of first registration (February 11, 2019).

## Introduction

Colorectal cancer (CRC) is the leading cancer worldwide^1–4^. Approximately 20% of patients with CRC present with stage IV (metastatic CRC (mCRC)), and approximately 80% present with stage I–III. Unfortunately, approximately 40% of patients with stage I–III finally progress to mCRC^5^. Despite the advancement of biochemotherapy for the treatment of mCRC, the median overall survival (OS) remains at 30–36 months^6–10^. Before the availability of new agents, the more precise strategy in selecting the right biochemotherapy for the right patients remains the cornerstone for mCRC treatment.

Treatment of mCRC may consist of chemotherapy, precision cancer medicines, and immunotherapy or some combination that is often determined by genomic testing of the cancer^2,6,9–17^. Some patients might be only treated with precision cancer agents or immunotherapy^16,17^ and might avoid chemotherapy at initial treatment. Systemic agents for mCRC consist of chemotherapy based on a fluoropyrimidine (5-FU), oxaliplatin, and irinotecan (in combination or in sequence) and monoclonal/targeted agents targeting BRAF mutation^18–20^, NTRK fusion oncoprotein^21,22^, vascular endothelial growth factor (VEGF) and epidermal growth factor receptor (EGFR) (in patients with RAS and BRAF wild-type tumors)^23,24^ as well as anti-programmed death-1 (PD-1)/ anti-programmed death ligand-1 (PD-L1) agents targeting patients with microsatellite instability-high (MSI-H) or with high tumor mutation burden^16,17^. Unfortunately, majority of patients finally develop drug resistance and thereby require late-line agents such as regorafenib^25,26^ and TAS-102^27–29^.

These two agents are the late-line therapeutic options for patients. Sadly, the PR rate of both regorafenib and TAS-102 is approximately 1%–3%, comprising 60% of patients who did not respond to these agents. To date, several studies^6,25–28,30–33^ try to prove the sequence of regorafenib and TAS-102 for the treatment of refractory mCRC, and majority of them point out that their sequences did affect the OS^33–36^. However, a key difference was observed between regorafenib and TAS-102. TAS-102 is a traditional chemotherapy^37–39^ and regorafenib^15,25,26,29,31,40,41^ is a multiple oncogenic receptor tyrosine kinases inhibitor^25,26,30,31,42^. In a systematic review and network meta-analysis comparing regorafenib and TAS-102 showed similar OS and progression-free survival (PFS). In the subgroup analysis, regorafenib was associated with a lower all-grade toxicity for anemia, neutropenia and thrombocytopenia. On contrast, regorafenib was associated with higher all-grade hand foot skin reaction (HFSR). In addition, regorafenib was associated with a lower grade 3 to 5 toxicity for anemia and neutropenia, but regorafenib showed a higher grade 3–5 HFSR and fatigue compared with TAS-102^33^. Its efficacy was further proven in the IMblaze370 study, showing that atezolizumab with or without cobimetinib versus regorafenib in a previously treated mCRC did not differ^43^. Thus, the question is not the efficacy of regorafenib, but who is the right patient for regorafenib. To the best of our knowledge, no consensus or strategy exists in selecting the right patient for regorafenib administration.

In our multicenter retrospective study in Taiwan, a large-scale cohort was used to explore the prognostic markers of refractory mCRC after the regorafenib treatment. Significant prognostic markers were used to determine an easy and more precise guide to select the right patients for regorafenib, leading to more survival and avoiding unnecessary HFSR.

## Results

### Patient characteristics

Patients’ baseline characteristics are shown in Table 1. A total of 613 patients were enrolled for analysis. The mean age of patients was 61.4 years, and the mean body mass index was 23.4 kg/m^2^. About 76.3% of patients had good performance status (Eastern Cooperative Oncology Group (ECOG) 1 and 2), 2.4% had not only one location of primary tumor, and 77.8% had a left-sided mCRC. About 59.4% of patients had more than one metastatic site. In patients with available gene testing, the mutation rates of KRAS, NRAS, BRAF, and MSI-H were 46.5%, 5.9%, 2.9%, and 3.1%, respectively.

**Table 1:** Patients’ characteristics (n = 613).

	n	(%)
Age (y/o) ± (SD)	61.4	(11.7)
BMI ± (SD)	23.4	(4.3)
Gender		
Female	262	(42.7)
Male	351	(57.3)
Performance status (ECOG)		
0	303	(49.4)
1	165	(26.9)
2	91	(14.8)
3	54	(8.8)
Stage (AJCC 6th)		
I	15	(2.4)
II	50	(8.2)
III	159	(25.9)
IV	389	(63.5)
Location		
Left	477	(77.8)
Multi-location	15	(2.4)
Right	121	(19.7)
Histology		
Adenocarcinoma	592	(96.6)
Mucinous adenocarcinoma	17	(2.8)
Carcinoma	4	(0.7)
Primary lesion resection		
No	87	(14.2)
Yes	525	(85.8)
Number of metastases		
1	249	(40.6)
2	243	(39.6)
3	109	(17.8)
4	11	(1.8)
5	1	(0.2)
CEA > = 50 (ng/mL)		
No	327	(53.3)
Yes	286	(46.7)
KRAS status		
Wild	317	(51.7)
Mutant	275	(44.9)
NA	21	(3.4)
NRAS status		
Wild	190	(31.0)
Mutant	12	(2.0)
NA	411	(67.0)
BRAF status		
Wild	199	(32.5)
Mutant	6	(1.0)
NA	408	(66.6)
Microsatellite status		
MSS	247	(40.3)
MSI-H	8	(1.3)
NA	358	(58.4)

AJCC, American Joint Committee on Cancer; BMI, body mass index; CEA, carcino embryonic antigen; ECOG, Eastern Cooperative Oncology Group; NA, non-available; MSI-H, microsatellite instability-high; MSS, microsatellite stable; SD, standard deviation.

### Treatment efficacy

**Figure 1:**
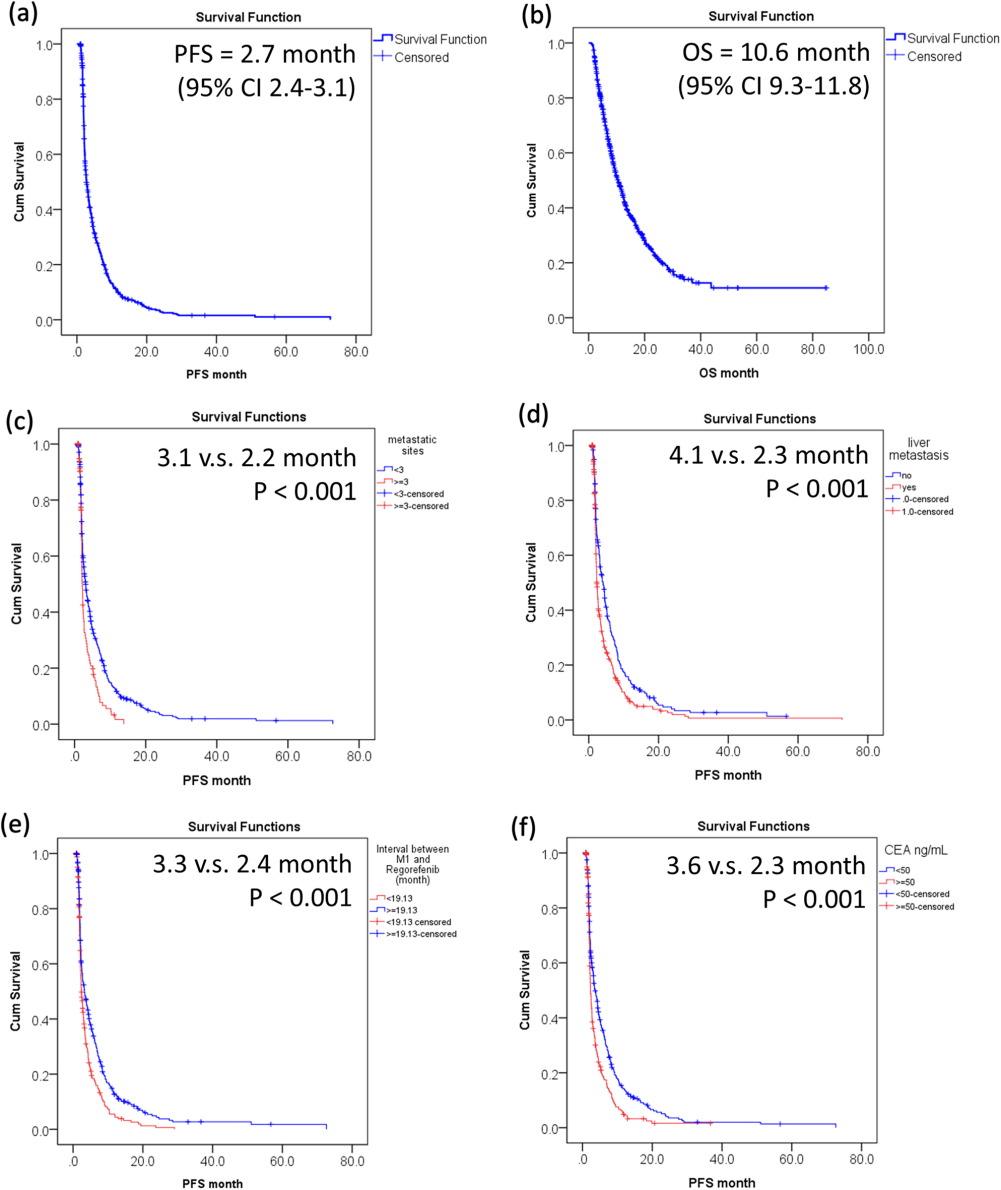
Kaplan–Meier curves for (**a**) progression-free survival (PFS); (**b**) overall survival (OS); (**c**) PFS by metastatic sites; (**d**) PFS by liver metastasis; (**e**) PFS by interval between M1 and regorafenib; (**f**) PFS by Carcino Embryonic Antigen (CEA) level.

Patients administered with regorafenib had a median PFS of 2.7 months (95% confidence internal (CI), 2.4–3.1) (Fig. 1a). Patients administered with regorafenib had a median OS of 10.6 (95% CI, 9.3–11.8) months (Fig. 1b). The response rate is shown in Supplemental Table 1. For the overall study population, PR, SD, progressive disease (PD) and non-assessable rates were 2.4% (n = 15), 36.4% (n = 223), 53.8% (n = 330), and 7.3% (n = 45), respectively. The impact of the initial regorafenib dosage on response is listed in Supplemental Table 2, the initially standard dosage did not significantly impact on regorafenib efficacy. The disease control rate (DCR) is not different between those with < 160 mg than with 160 mg (40.2% vs. 37.9%).

### Prognostic factors for PFS and OS from univariate and multivariate analyses

**Figure 2:**
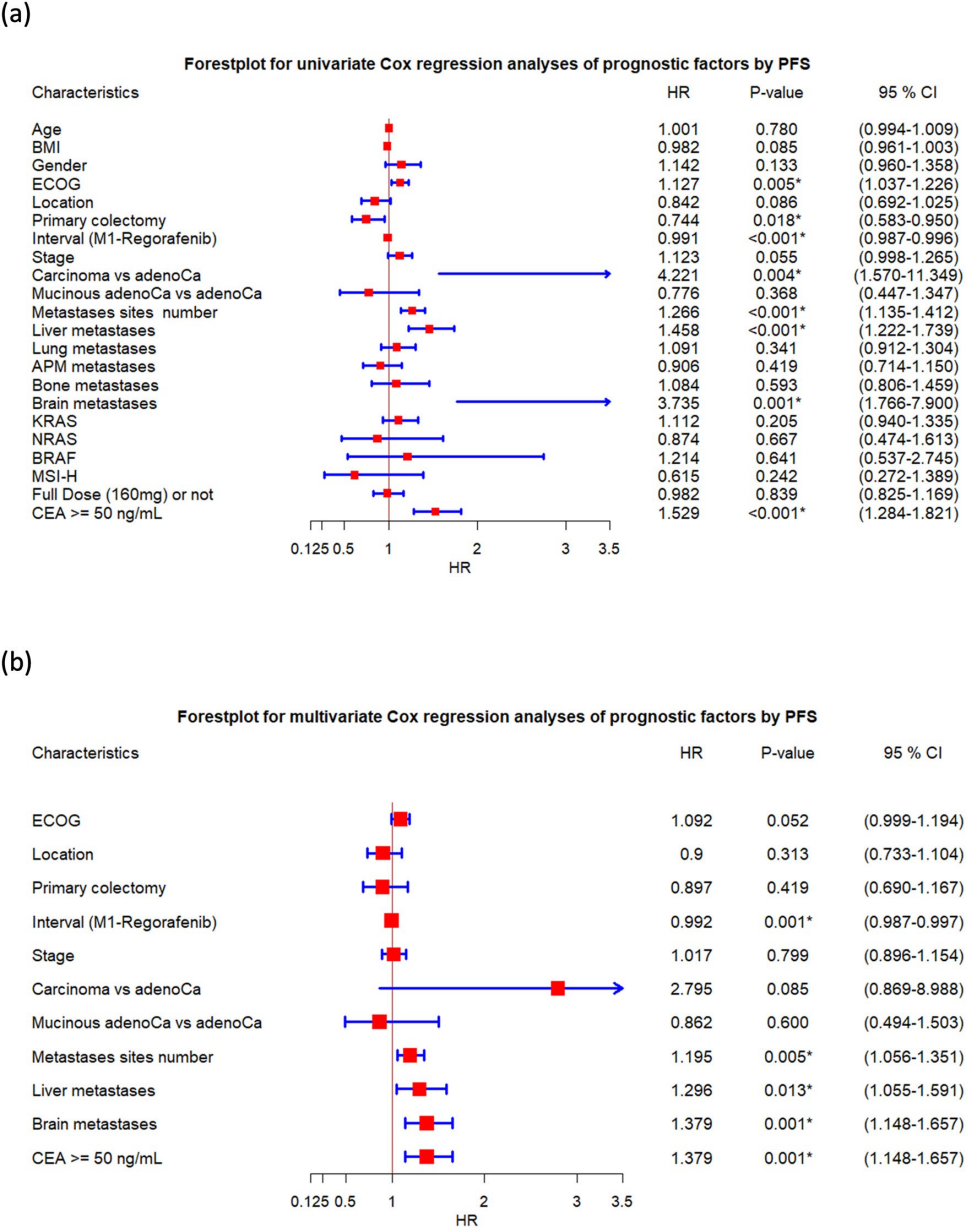
The forest plots summarize the (**a**) univariate and (**b**) multivariate analyses of prognostic factors in predicting progression-free survival (PFS). APM, abdominal/peritoneal metastasis.

**Figure 3:**
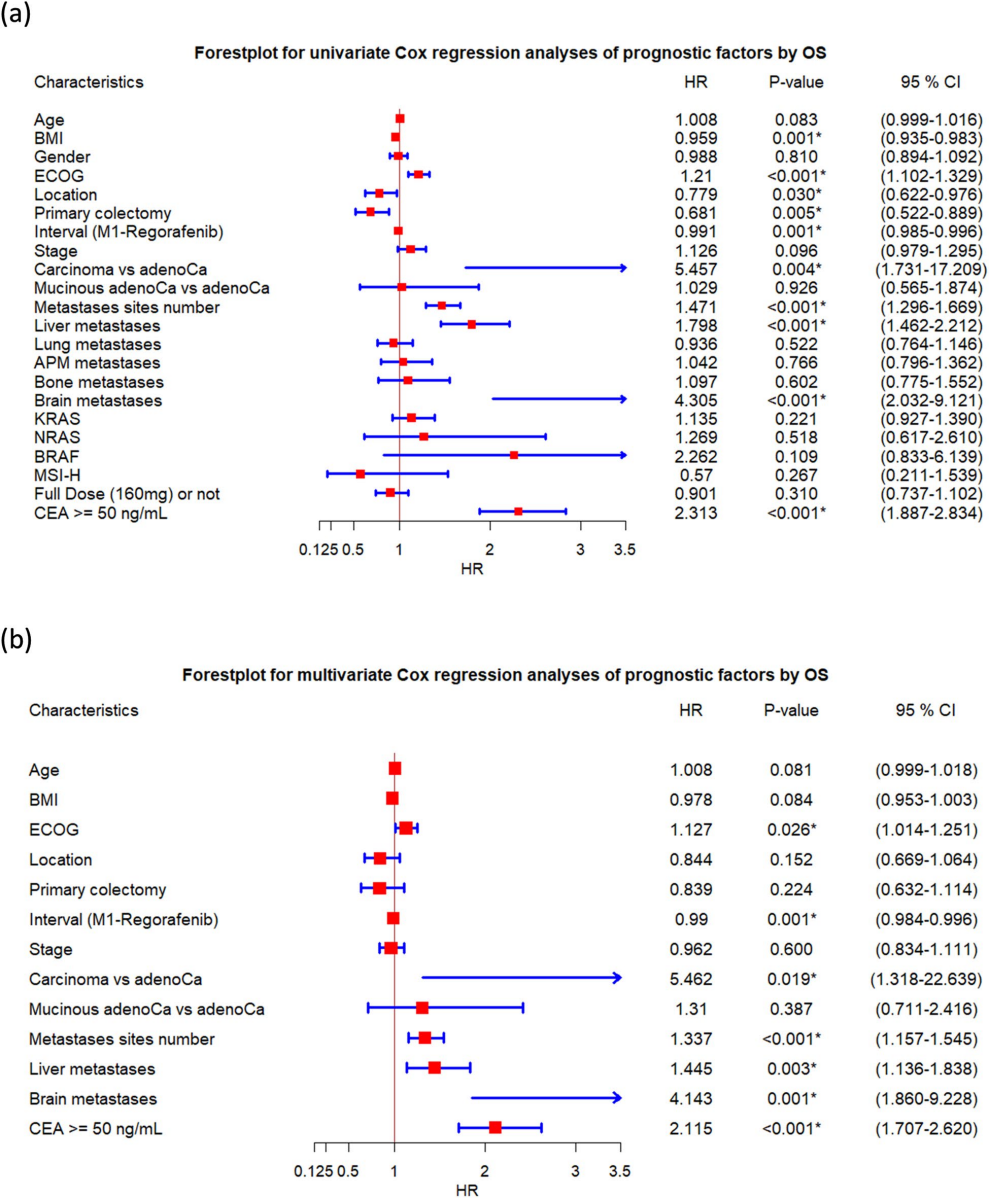
The forest plots present the (**a**) univariate and (**b**) multivariate analyses of prognostic factors in predicting overall survival (OS). Abbreviation: APM, abdominal/peritoneal metastasis.

To clarify the potential prognostic factors in the treatment of refractory mCRC by regorafenib, a Cox proportional hazards model was used. The forest plot in Fig. 2 summarizes the univariate (Fig. 2a) and multivariate (Fig. 2b) analyses of prognostic factors to predict the PFS (The data is shown in Supplemental Table 3). The forest plot in Fig. 3 presents univariate (Fig. 3a) and multivariate (Fig. 3b) analyses of prognostic factors in predicting OS (The data is shown in Supplemental Table 4).

In univariate analysis according to PFS, ECOG, primary colectomy, interval between M1 and regorafenib, pathology (Carcinoma vs adenocarcinoma), number of metastatic sites, liver metastasis, brain metastasis, and CEA high (≥ 50 ng/mL) were identified as significant prognostic factors in predicting PFS. After controlling for other potential confounding factors, interval between M1 and regorafenib, number of metastatic sites, liver metastasis, brain metastasis, and CEA high (≥ 50 ng/mL) remained as independent prognostic factors. The reprehensive KM survival plots by independently significant factors are shown in Fig. 1. Patients with ≥ 3 metastatic sites had a poor PFS than those with < 3 metastatic sites (2.2 vs. 3.1 months, P < 0.001) (Fig. 1c). Patients without liver metastasis had a better PFS than those with liver metastasis (4.1 vs. 2.3 months, P < 0.001) (Fig. 1d). Patients with interval between metastasis (M1) and regorafenib of ≥ 19.13 months had a good PFS than those with < 19.13 months (3.3 vs. 2.4 months, P < 0.001) (Fig. 1e). Patients with CEA of ≥ 50 ng/mL had poor PFS than those with lower CEA levels (2.3 vs. 3.6 months, P < 0.001) (Fig. 1f).

In univariate analysis according to OS, BMI, ECOG, location, primary colectomy, interval between M1 and regorafenib, pathology (carcinoma vs adenocarcinoma), number of metastatic sites, liver metastasis, brain metastasis, and CEA high (≥ 50 ng/mL) were identified as significant prognostic factors in predicting OS. After controlling for other potential confounding factors, ECOG, interval between M1 and regorafenib, pathology (carcinoma vs adenocarcinoma), number of metastatic sites, liver metastasis, brain metastasis, and CEA high (≥ 50 ng/mL) remained as independent prognostic factors.

### The clustering heatmap

**Figure 4:**
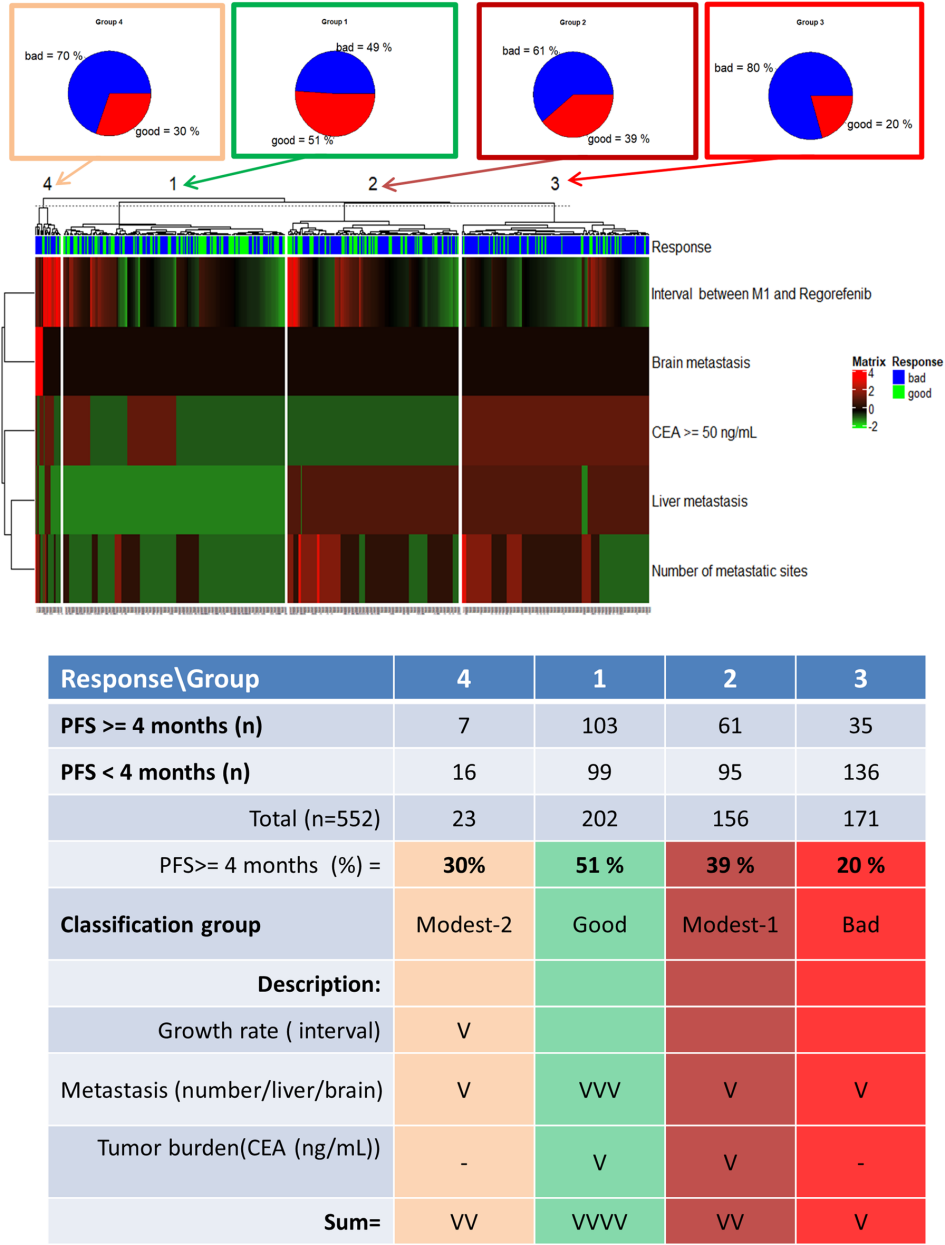
The clustering heatmap (k-means = 4) was used to classify patients into good, bad, modest 1, and modest 2 groups according to significantly independent prognostic factors by PFS >  = 4 months or not. (Top) Pie charts represent the distribution of patients with good and bad responses in four groups. (Middle) Clustering heatmap visualizes the different groups’ distribution of independent prognostic factors. (Bottom) Independent prognostic factors were classified into three patterns of cancer behaviors: growth rate, metastasis ability, and tumor burden. It was used to score the grouped patients. Abbreviations: ad, adenocarcinoma; HR, hazard ratio; CI, confidence interval.

In the attempt to classify patients taking regorafenib into different groups according to response, a total of 552 patients with PFS of ≥ 4 months or those with event (PD) of < 4 months were used to create the clustering heatmap. The rationale in selecting the cut-off level of 4 months with scientific aspect based on median PFS of 2 months in CORRECT trial^25^. It means patient apply twice, indicating a true response to regorafenib. Then, the response was good if PFS was ≥ 4 months and bad if PFS was < 4 months. Significant prognostic factors in multivariate Cox proportional hazards model analysis were used in the clustering heatmap (2). Both the row and column clustering were arranged. The k-means cluster is 4. The clustering heatmap is shown in the middle of Fig. 4. Patients were grouped into three major groups: good, modest, and bad. The modest group is further classified into modest-1 and modest-2 groups. The PFS >  = 4 months rates in the good and bad groups were 51% and 20%, respectively. The PFS >  = 4 months rates in the modest-1 and modest-2 groups are 39% and 30%, respectively. The statical results are shown at the top and bottom in Fig. 4. The characteristics of patients in the bad group were large burden (CEA >  = 50 ng/mL) and rapid growth (short interval between M1 and regorafenib) and poor/scanty spread (multiple metastatic sites and liver metastasis). The carton-like score used to demonstrate differences among groups is listed at the bottom in Fig. 4.

### Decision tree analysis to develop predictive algorithms

**Figure 5:**
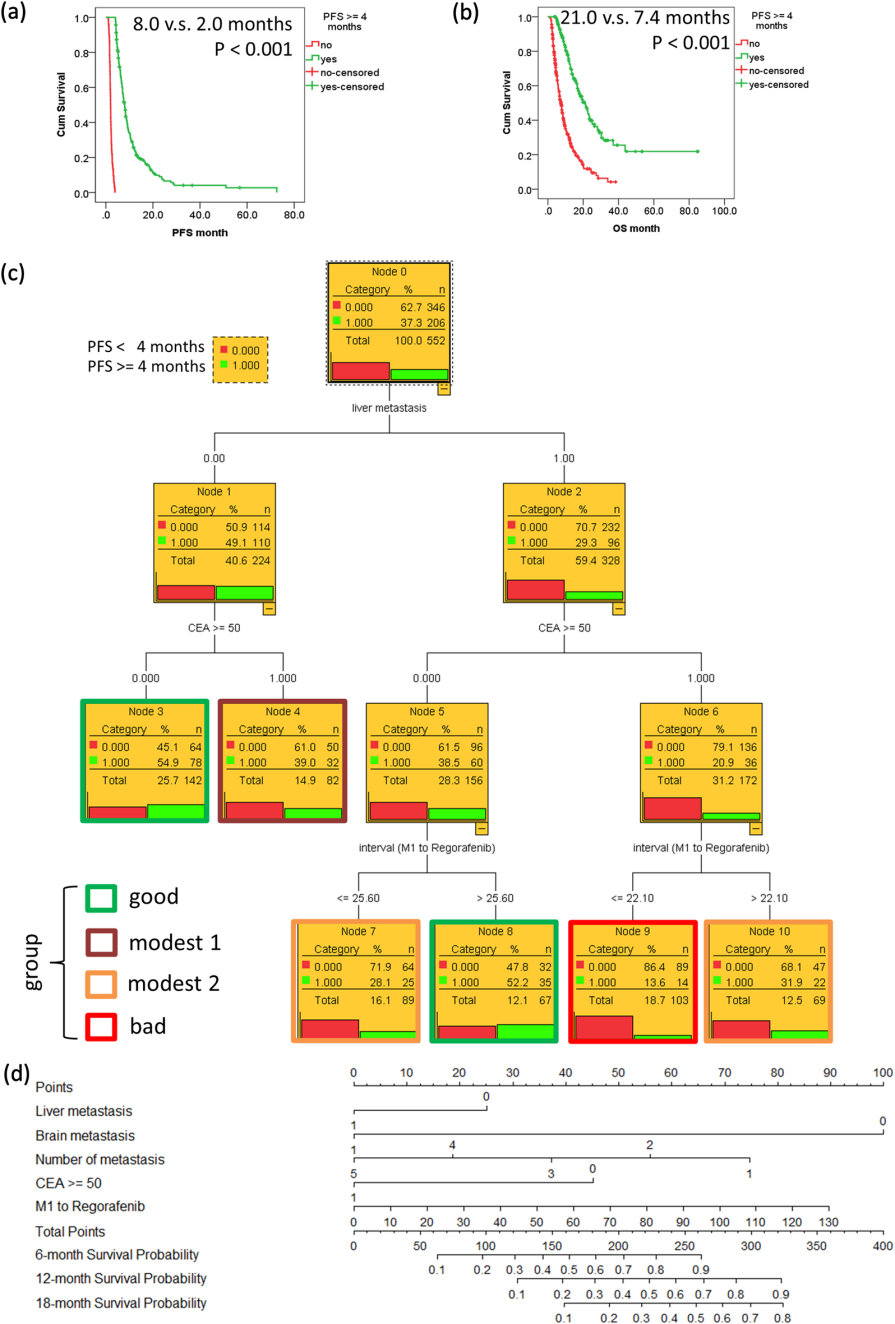
Kaplan–Meier curves for (**a**) progression-free survival (PFS); (**b**) overall survival (OS) by patient with PFS >  = 4 months or not. (**c**) The decision tree in predicating the priority right of regorafenib for the treatment of refractory metastatic colorectal cancer according to liver metastasis, CEA, and interval between M1 and regorafenib. (**d**) The prognostic nomogram for OS of patients after regorafenib.

The critical role of patients with PFS >  = 4 months is shown in Fig. 5a,b. Patients with PFS >  = 4 months significantly had good PFS and OS, comparing to those with PFS < 4 months. The decision tree based on Chi-square Automatic Interaction Detector (CHARD) was used to develop the predictive algorithms. A total of 552 patients with PFS >  = 4 months or those with event (PD) at < 4 months was used to create a decision tree (Fig. 5c) (Decision tree with variable statistics is listed in supplemental Fig. 1). The significant prognostic factors for PFS in multivariate Cox proportional hazards model analysis were used in the decision tree. Patients were divided into four groups. The same term was used to describe these groups: good, modest-1, modest-2, and bad. The PFS >  = 4 months rates in the good, modest-1, modest-2 and bad group were 54.1% ((78 + 35)/ (142 + 67)), 39.0%, 30.0% ((22 + 25)/ (69 + 89)) and 13.6%, respectively. The PFS >  = 4 months rate in the decision tree is similar to that of the clustering heatmap. However, it is can be easily incorporated into clinical practice.

### Prognostic nomogram

Our previous algorithms would be partially validated for OS. The prognostic nomogram for OS of patients with mCRC after regorafenib was performed (Fig. 5d) (The 12-month calibration curve of the nomogram is shown in supplemental Fig. 2). In the nomogram for OS, each independent prognostic markers with a given value can be mapped to the points axis. The sum of these points can be referred to in the total points axis. The sum of these numbers is located on the total points axis, and a line is drawn downward to the survival axis to determine the likelihood of 6-month, 12 month and 18-month OS, respectively. The c-index was 0.674 and it was acceptable.

## Discussion

To our knowledge, this is the first study that creates a strategy in selecting the right patients for the treatment of refractory mCRC with regorafenib using a large-scale post-market data. The flowchart representing a decision tree and prognostic nomogram are shown in Fig. 5c. Regorafenib could be considered in patients without liver metastasis and CEA of < 50 ng/mL or in those with liver metastasis, CEA < 50 ng/mL and interval between M1 and regorafenib > 25.6 months. Conversely, regorafenib might not be used with top priority in patients with liver metastasis, CEA of >  = 50 ng/mL and interval between M1 and regorafenib of < 22.1 months.

The rationale for our approaching strategy based on the results of clarifying the potential prognostic factors for PFS via the univariate and multivariate Cox proportional hazards model for the treatment of refractory mCRC by regorafenib. The clustering heatmap visualization helps us understand the rationale. The end-point is the PFS of ≥ 4 months and patients were classified into good and bad responses (PFS >  = 4 months vs PFS < 4 months). In Fig. 4, regorafenib could be considered in patients with low tumor growth rate (long interval between M1 and regorafenib), lower metastatic ability (less metastatic sites and without liver or brain metastasis), and low tumor burden (low CEA level). These patients were the good group, and the 4-month PFS rate is 51%. Conversely, regorafenib might not be indicated for patients with high tumor growth rate (short interval between M1 and regorafenib), high metastatic ability (more metastatic sites and/or with liver metastasis), and high tumor burden (high CEA level). These patients were in the bad group with a 4-month PFS rate of 20%. The 2.5-fold change of response rate was noted among patients in the good group as compared to those in the bad group. In addition, patients in the modest-1 and modest-2 groups have 4-month PFS rate of 39% and 30%, respectively. Then, the flowchart representing the decision tree and prognostic nomogram are easily applicable into clinical practice (Fig. 5c,d). Moreover, RAS and BRAF were not considered as prognostic markers, which are similar to those of previous literatures^25,44^. Moreover, 255 of 613 enrolled patients had microsatellite status reports, and the incidence of MSI-H was 3.1% and MSI-H status is not a significant prognostic factor in PFS and OS analyses. As compared with previous studies (subgroups analysis of CORRECT^25^ AND CONCUR^26^), the time from the first diagnosis of metastatic disease to randomization did not affect the efficacy of regorafenib, which is not similar to our findings. However, (K-)RAS and BRAF states did not interfere the regorafenib efficacy and were similar to our findings.

In our study, the median PFS and OS in patients taking regorafenib were 2.7 (95% CI, 2.4–3.1) and 10.6 (95% CI, 9.3–11.8) months, respectively. The PFS is similar to those of previous studies; however, the OS was longer than that of patients taking regorafenib in the CORRECT trial in which patients had a median OS of 6.4 (PFS, 1.9) months^25^ and in the REBECCA trial in which patients had a median OS of 5.5 (PFS, 2.7) months^30^. The CORRELATE trial, a real-world analysis of regorafenib effectiveness, showed a median OS of 7.6 months^42^. A trend toward longer OS in the late-run clinical studies was observed (CORRECT in 2013; REBECCA in 2016; CORRELATE in 2017 and our study in 2020). The longer OS observed in our study may be attributed to the addition of other salvage regimen, such as TAS-102 and challenge of previously active agents. A subgroup analysis of Taiwanese patients with mCRC in the CORRELATE trial showed a median OS of 11.6 months in 2018^45^.

With the respect of an impact of dosage on regorafenib efficacy, the result is listed in the Supplemental Table 2. The initial dosage of 160 mg or not did not affect the PR and DCR rates. Our report is similar to those of previous studies. The initially standard dosage did not significantly impact on regorafenib efficacy. The PR and DCR were not different between those with < 160 mg than with 160 mg (PR: 2.8% vs. 2.0% and DCR: 37.9% vs. 40.2%). Similarly, with those in ReDOSE study^31^ studies, an escalation strategy has previously been shown to improve survival as compared with those of a standard-dose regimen.

In the previous papers about survival of peritoneal metastasis^46,47^, most of them reported that the survival of peritoneal metastasis was poor. In our study, abdominal/peritoneal metastasis (APM) was not a prognostic marker in the univariate analysis according to PFS and OS. The response rate was also not different between patients with APM or not (P-value = 0.407). For patients with APM, rates of PR, SD or PD were 1.1%, 44.3% and 54.5%, respectively. For patients without APM, rates of PR, SD, and PD were 2.9%, 38.3%, and 58.8%, respectively. In the PRODIGE 7 trial, the median OS was 41.7 months in the cytoreductive surgery plus hyperthermic intraperitoneal chemotherapy group and 41.2 months in the cytoreductive surgery group (hazard ratio 1.00; stratified log-rank P-value = 0.99)^48^. It pointed out that selected patients with APM had the relative better OS. A reasonable explanation for our finding was that some patients with APM might die early in the course of disease. Patients who could receive regorafenib were somehow a relatively good subpopulation. The peritoneal cancer index score was a good tool in predicting the impact of peritoneal metastases burden on survival. The further study should be arranged to clarify the impact of peritoneal metastases burden on efficacy of regorafenib.

Several limitations were observed in our study. First, its retrospective design may have led to an inevitable selection bias. Second, patients who did not continued the treatment for > 3 weeks were excluded in the analysis during screening also leading to selection bias, e.g., patients with an intrinsically better prognosis were selected. Third, adverse effects reports were not provided, because not all patients in the study had detailed documented adverse effects. Fourth, patient’s preferred dosage or sequence with TAS-102 was also part of physician’s consideration when determining the treatment sequence and dosage. Finally, TAS-102 reimbursement was only partially permitted in Taiwan during the later parts of this study and most of patients did not receive TAS-102 before the use of regorafenib. Therefore, our proposed algorithm for selecting patients who will benefit most from regorafenib would be only applicated in patients with drug resistance to 5-FU, irinotecan, oxaliplatin, bevacizumab, and cetuximab/panitumumab (in RAS wild-type patients).

## Conclusion

Regorafenib could be considered in patients without liver metastasis and CEA of < 50 ng/mL or in those with liver metastasis, CEA < 50 ng/mL and interval between M1 and regorafenib > 25.6 months. Conversely, regorafenib might not be used with top priority in patients with liver metastasis, CEA of >  = 50 ng/mL and interval between M1 and regorafenib of < 22.1 months.

## Material and methods

### Patients

This was a multicenter retrospective cohort study based on patient data collected between August 2012 and January 2018 at Chang-Geng Medical Foundation Linkou Chang-Geng Memorial Hospital, Koo Foundation Sun Yat-Sen Cancer Center, and Taipei Veterans General Hospital, Taiwan. This study followed the Helsinki Declaration guidelines and was approved by the ethics committees and institutional review boards of the respective institutions. The requirement for informed consent was waived due to the retrospective nature of the study.

Patients with pathologically confirmed refractory mCRC and had been administered regorafenib for > 3 weeks were enrolled into the analysis. They were resistant to 5-FU, irinotecan, oxaliplatin, bevacizumab, and cetuximab/panitumumab (in RAS wild-type patients). Basic clinicopathologic parameters were recorded, including age, gender, tumor location, pathologic features (e.g., histological type, grade, RAS, BRAF, and MSI), and metastatic sites. The definition of APM generally refers to the metastatic involvement of the peritoneum. The right-sided CRC consist of cecum, ascending colon, hepatic flexure, and transverse colon and the left-sided CRC consist of splenic flexure, descending colon, sigmoid colon, rectosigmoid junction, and rectum. Both of the standard regimen (160 mg) or escalation strategy (< 160 mg) for initial regorafenib treatment were frequently implemented depending on the physician’s judgment and based on patient compliance and adverse effects.

### Efficacy evaluation

The metastatic disease was evaluated based on the Response Evaluation Criteria in Solid tumor version 1.1. The best response was recorded after the treatment initiation, and the disease control rate was regarded as the proportion of patients with complete response (CR), partial response (PR), and stable disease (SD). We investigated OS (the time from regorafenib to the time of death caused by the disease) and PFS (the time from regorafenib to the time of disease progression observed by radiologic imaging or the time when intolerable adverse effects were observed).

### Statistical analysis

Continuous data are expressed as mean ± standard deviation. Statistical comparisons were based on nonparametric tests. Correlations between clinicopathological variables and responses were analyzed using χ^2^ test or Fisher’s exact test. Survival was estimated using the Kaplan–Meier (KM) method. The Cox proportional hazards model was used for univariate and multivariate analyses to determine the prognostic impact on clinicopathological factors on survival end-points. Factors with *P*-value of < 0.10 in the univariate analysis were enrolled in the multivariable analysis. Significant prognostic factors in multivariate analyses using a Cox proportional hazards model were enrolled into the decision tree analysis. The forest plot was used to visualize the results.

The R program and complex heatmap package^49^ were used to generate clustering heatmap from significant prognostic factors in multivariate analyses using a Cox proportional hazards model. Good response group was defined as patients with PFS of ≥ 4 months, whereas bad response as patients with PD event and PFS of < 4 months. This clustering heatmap with k-means of 4 was used to visualize the clinical pattern of patients with good and bad responses to regorafenib^49^. The clustering was applied to grouping patients according to independent significant prognostic factors in multivariate analyses according to PFS. The decision tree with Chi-Square Automatic Interaction Detector model was drawn according to same independent significant prognostic factors in multivariate analyses according to PFS. The prognostic nomogram for OS of patients with mCRC after regorafenib was performed by using R software.

A two-sided *P*-value of < 0.05 was regarded as statistically significant. SPSS software was used for all statistical analyses, and the R program was also used.

### Ethical approval

This retrospective study was conducted based on population-based data from Chang-Geng Medical Foundation Linkou Chang-Geng Memorial Hospital, Koo Foundation Sun Yat-Sen Cancer Center, and Taipei Veterans General Hospital, Taiwan. This study followed the Helsinki Declaration guidelines and was approved by “Chang Gung Medical Foundation Institutional Review Board”,” Institutional Review Board of the Koo Foundation Sun Yat-Sen Cancer Center” and “Institutional Review Board of the Taipei Veterans General Hospital”. The requirement for informed consent was waived due to the retrospective nature of the study by “Chang Gung Medical Foundation Institutional Review Board”,” Institutional Review Board of the Koo Foundation Sun Yat-Sen Cancer Center”, and “Institutional Review Board of the Taipei Veterans General Hospital”.

## Supplementary Information

Supplementary Information.
