# Supplementary material for: Preference criteria for regorafenib in treating refractory metastatic colorectal cancer are the small tumor burden, slow growth and poor/scanty spread
Source: Sci Rep. 2021 Jul 28;11:15370. doi: 10.1038/s41598-021-94968-x (PMC8319410; doi:10.1038/s41598-021-94968-x)

**Original article**

**Preference criteria for regorafenib in treating refractory metastatic colorectal cancer are the** **small tumor burden, slow growth and poor/scanty spread.**

Hung-Chih Hsu, M.D., Kuo-Cheng Huang,M.D., Wei-Shone Chen,M.D., [Jeng-Kai Jiang](http://link.springer.com/search?facet-author=%22Jeng-Kai+Jiang%22),M.D., [Shung-Haur Yang](http://link.springer.com/search?facet-author=%22Shung-Haur+Yang%22),M.D., Huann-Sheng Wang,M.D., Shih-Ching Chang,M.D., Yuan-Tzu Lan,M.D., Chun-Chi Lin,M.D., Hung-Hsin Lin,M.D., Sheng-Chieh Huang,M.D., Hou-Hsuan Cheng, M.D., Tsai-Sheng Yang, M.D., Chien-Chih Chen , M.D., Yee Chao, M.D., Hao-Wei Teng* M.D.&Ph.D.

| **Supplemental Table 1.** The response distribution in patients treated with regorafenib. | | | | | | | | |
| --- | --- | --- | --- | --- | --- | --- | --- | --- |
|  | PR | | SD | | PD | | NA | |
|  | n | (%) | n | (%) | n | (%) | n | (%) |
| Response Rate | 15 | (2.4) | 223 | (36.4) | 330 | (53.8) | 45 | (7.3) |
| **Abbreviations:** PD, progressive disease; PR, partial response; SD, stable disease, NA, not assessable. | | | | | | | | |

| **Supplemental Table 2.** The response distribution by initial starting dosage of regorafenib. | | | | | | | | | |
| --- | --- | --- | --- | --- | --- | --- | --- | --- | --- |
|  | | Response | | | | | | | |
|  |  | PR | | SD | | PD | | NA | |
|  |  | n | (%) | n | (%) | n | (%) | n | (%) |
| Initial Dosage (mg/day) | .<160 | 10 | 2.8% | 126 | 35.1% | 196 | 54.6% | 27 | 7.5% |
|  | 160 | 5 | 2.0% | 97 | 38.2% | 134 | 52.8% | 18 | 7.1% |
| **Abbreviations:** PD, progressive disease; PR, partial response; SD, stable disease, NA, not assessable. | | | | | | | | | |

| **Supplemental Table 3**. Univariate and multivariate Cox regression analyses of prognostic factors for PFS in patients with colorectal cancer (n=613). | | | | | | | |
| --- | --- | --- | --- | --- | --- | --- | --- |
|  | Univariate | | |  | Multivariate | | |
|  | P-value | HR | (95.0% CI) |  | P-value | HR | (95.0% CI) |
| Age | 0.780 | 1.001 | (0.994-1.009) |  | - | - | - |
| BMI | 0.085 | 0.982 | (0.961-1.003) |  | - | - | - |
| Gender | 0.133 | 1.142 | (0.960-1.358) |  | - | - | - |
| ECOG | 0.005* | 1.127 | (1.037-1.226) |  | 0.052 | 1.092 | (0.999-1.194) |
| Location | 0.086 | 0.842 | (0.692-1.025) |  | 0.313 | 0.900 | (0.733-1.104) |
| Primary colectomy | 0.018* | 0.744 | (0.583-0.950) |  | 0.419 | 0.897 | (0.690-1.167) |
| Interval (M1-Regorafenib) | <0.001* | 0.991 | (0.987-0.996) |  | 0.001* | 0.992 | (0.987-0.997) |
| Stage | 0.055 | 1.123 | (0.998-1.265) |  | 0.799 | 1.017 | (0.896-1.154) |
| Pathology |  |  |  |  |  |  |  |
| Carcinoma vs adenoCa | 0.004* | 4.221 | (1.570-11.349) |  | 0.085 | 2.795 | (0.869-8.988) |
| Mucinous adenoCa vs adenoCa | 0.368 | 0.776 | (0.447-1.347) |  | 0.600 | 0.862 | (0.494-1.503) |
| Metastases sites number | <0.001* | 1.266 | (1.135-1.412) |  | 0.005* | 1.195 | (1.056-1.351) |
| Liver metastases | <0.001* | 1.458 | (1.222-1.739) |  | 0.013* | 1.296 | (1.055-1.591) |
| Lung metastases | 0.341 | 1.091 | (0.912-1.304) |  | - | - | - |
| APM metastases | 0.419 | 0.906 | (0.714-1.150) |  | - | - | - |
| Bone metastases | 0.593 | 1.084 | (0.806-1.459) |  | - | - | - |
| Brain metastases | 0.001* | 3.735 | (1.766-7.900) |  | 0.001* | 1.379 | (1.148-1.657) |
| KRAS | 0.205 | 1.112 | (0.940-1.335) |  | - | - | - |
| NRAS | 0.667 | 0.874 | (0.474-1.613) |  | - | - | - |
| BRAF | 0.641 | 1.214 | (0.537-2.745) |  | - | - | - |
| MSI-H | 0.242 | 0.615 | (0.272-1.389) |  | - | - | - |
| Full Dose(160mg) or not | 0.839 | 0.982 | (0.825-1.169) |  | - | - | - |
| CEA >= 50 ng/mL | <0.001* | 1.529 | (1.284-1.821) |  | 0.001* | 1.379 | (1.148-1.657) |
| **Abbreviations**: AJCC, American Joint Committee on Cancer; Adenocarcinoma Carcinoma, AdenoCa; APM, abdominal peritoneal metastasis; LVSI, lymph-vascular space invasion; MSI-H, microsatellite instability high; PD-L1, programmed cell death ligand-1; SD, standard deviation; TCs, tumor cells, TILs, tumor-infiltrating lymphocytes  *: significant , P value <0.1 entry to multiple | | | | | | | |

| **Supplemental Table 4**. Univariate and multivariate Cox regression analyses of prognostic factors for OS in patients with colorectal cancer (n=613). | | | | | | | |
| --- | --- | --- | --- | --- | --- | --- | --- |
|  | Univariate | | |  | Multivariate | | |
|  | P-value | HR | (95.0% CI) |  | P-value | HR | (95.0% CI) |
| Age | 0.083 | 1.008 | (0.999-1.016) |  | 0.081 | 1.008 | (0.999-1.018) |
| BMI | 0.001* | 0.959 | (0.935-0.983) |  | 0.084 | 0.978 | (0.953-1.003) |
| Gender | 0.810 | 0.988 | (0.894-1.092) |  |  |  |  |
| ECOG | <0.001* | 1.210 | (1.102-1.329) |  | 0.026* | 1.127 | (1.014-1.251) |
| Location | 0.030* | 0.779 | (0.622-0.976) |  | 0.152 | 0.844 | (0.669-1.064) |
| Primary colectomy | 0.005* | 0.681 | (0.522-0.889) |  | 0.224 | 0.839 | (0.632-1.114) |
| Interval (M1-Regorafenib) | 0.001* | 0.991 | (0.985-0.996) |  | 0.001* | 0.990 | (0.984-0.996) |
| Stage | 0.096 | 1.126 | (0.979-1.295) |  | 0.600 | 0.962 | (0.834-1.111) |
| Pathology |  |  |  |  |  |  |  |
| Carcinoma vs adenoCa | 0.004* | 5.457 | (1.731-17.209) |  | 0.019* | 5.462 | (1.318-22.639) |
| Mucinous adenoCa vs adenoCa | 0.926 | 1.029 | (0.565-1.874) |  | 0.387 | 1.310 | (0.711-2.416) |
| Metastases sites number | <0.001* | 1.471 | (1.296-1.669) |  | <0.001* | 1.337 | (1.157-1.545) |
| Liver metastases | <0.001* | 1.798 | (1.462-2.212) |  | 0.003* | 1.445 | (1.136-1.838) |
| Lung metastases | 0.522 | 0.936 | (0.764-1.146) |  |  |  |  |
| APM metastases | 0.766 | 1.042 | (0.796-1.362) |  |  |  |  |
| Bone metastases | 0.602 | 1.097 | (0.775-1.552) |  |  |  |  |
| Brain metastases | <0.001* | 4.305 | (2.032-9.121) |  | 0.001* | 4.143 | (1.860-9.228) |
| KRAS | 0.221 | 1.135 | (0.927-1.390) |  |  |  |  |
| NRAS | 0.518 | 1.269 | (0.617-2.610) |  |  |  |  |
| BRAF | 0.109 | 2.262 | (0.833-6.139) |  |  |  |  |
| MSI-H | 0.267 | 0.570 | (0.211-1.539) |  |  |  |  |
| Full Dose(160mg) or not | 0.310 | 0.901 | (0.737-1.102) |  |  |  |  |
| CEA >= 50 ng/mL | <0.001* | 2.313 | (1.887-2.834) |  | <0.001* | 2.115 | (1.707-2.620) |
| **Abbreviations**: AJCC, American Joint Committee on Cancer; Adenocarcinoma Carcinoma, AdenoCa; APM, abdominal peritoneal metastasis; LVSI, lymph-vascular space invasion; MSI-H, microsatellite instability high; PD-L1, programmed cell death ligand-1; SD, standard deviation; TCs, tumor cells, TILs, tumor-infiltrating lymphocytes  *: significant , P value <0.1 entry to multiple | | | | | | | |

**Supplemental Figure 1**. Decision tree with variable statistics.

**
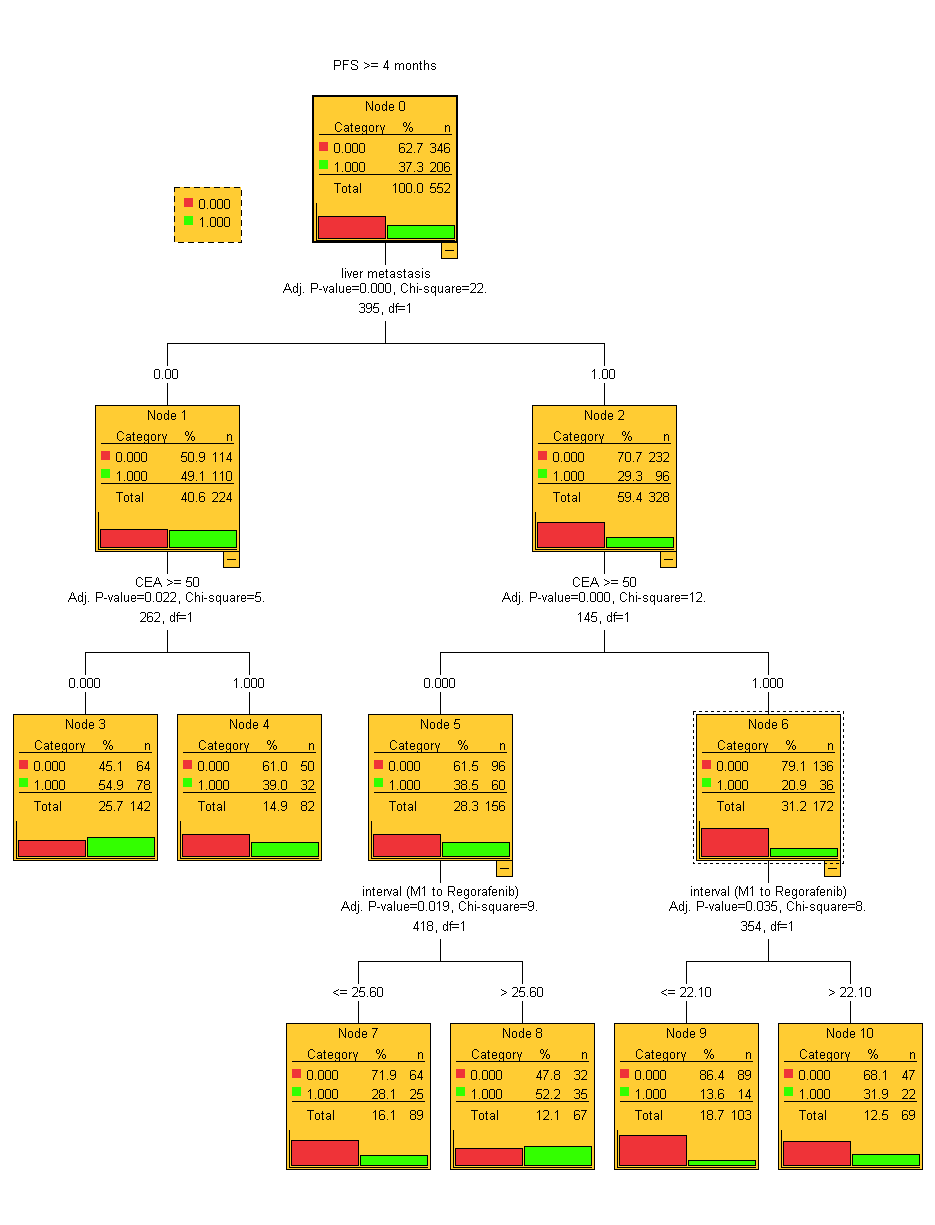
**

**Supplemental Figure 2**. The 12-month calibration curve of the nomogram.


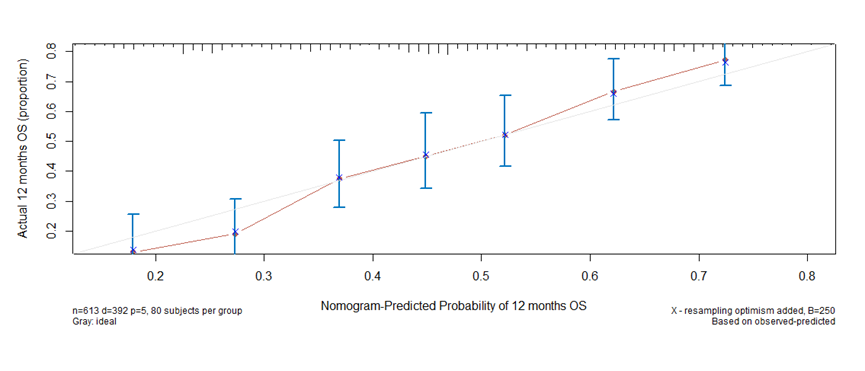

Supplement: Supplementary file 1 — Supplementary Information. [file 41598_2021_94968_MOESM1_ESM.docx]
